# SLC25A39 regulates Hedgehog signaling to promote tumor progression and sorafenib resistance in hepatocellular carcinoma

**DOI:** 10.1038/s41598-025-20008-7

**Published:** 2025-10-15

**Authors:** Qian Qiu, Hehe Yin, Lulu Zhang, Wen Xu, Shiwei Zhu, Qianqian Zhang, Mengqi Zhao, Yuting Qian, Yatong Ruan, Hui Zhang, Zihao Wu, Jiatao Liu, Jing Ke, Ying Dai, Wei Wang, Weijie Sun, Yufeng Gao, Honghai Xu

**Affiliations:** 1 Department of Pathology, the First Affiliated Hospital of Anhui Medical University, Hefei, 230022 China; 2 Department of Geriatrics, Division of Life Sciences and Medicine, The First Affiliated Hospital of USTC, University of Science and Technology of China, Hefei, 230002 China; 3 Anhui Key Laboratory of Geriatric Immunology and Nutrition Therapy, Hefei, 230027 China; 4 Department of Pharmacy, the First Affiliated Hospital of Anhui Medical University, Hefei, 230022 Anhui Province China; 5 Medical Oncology, The First Affiliated Hospital of Anhui Medical University, Hefei, 230022 China; 6 Department of Medical Oncology, First Affiliated Hospital of Bengbu Medical University, Bengbu, 233099 China; 7 Department of Infectious Diseases, the First Affiliated Hospital of Anhui Medical University, Hefei, 230022 China; 8 Anhui Province Key Laboratory of Infectious Diseases, Anhui Medical University, Hefei, 230022 China; 9 Present Address: Innovation and Entrepreneurship Laboratory for College Students, Anhui Medical University, Hefei, 230032 China

**Keywords:** Sorafenib, HCC, Hedgehog signaling pathway, SLC25A39, Acquired resistance, ScRNA-seq, Cancer, Tumour biomarkers

## Abstract

Sorafenib is the standard treatment for advanced hepatocellular carcinoma (HCC), yet resistance limits its efficacy. The Hedgehog (HH) signaling pathway contributes to drug resistance by maintaining HCC stem cell characteristics, but its role at the single-cell level is underexplored. Utilizing single-cell RNA sequencing (scRNA-seq) and machine learning, we identified solute carrier family 25 member 39 (SLC25A39) as a key regulator of the HH pathway. Analyses of various cohorts revealed that elevated SLC25A39 correlates with poor prognosis in HCC patients. SLC25A39 knockdown inhibited cancer stem cell characteristics and reduced sorafenib resistance in vitro. Furthermore, combined SLC25A39 suppression and sorafenib treatment significantly hindered HCC progression in both in vitro and in vivo models. These findings highlight the potential of shSLC25A39 to enhance sorafenib sensitivity , presenting a new avenue for combating drug resistance and enhancing therapeutic effectiveness.

**Supplementary Information:**

The online version contains supplementary material available at 10.1038/s41598-025-20008-7.

## Introduction

Primary liver cancer is the sixth most common cancer worldwide and the forth leading cause of cancer-related mortality^1^. Hepatocellular carcinoma (HCC) accounts for roughly 80% of all primary liver cancer cases. Due to the subtle nature of early symptoms, many patients are diagnosed at advanced stages, significantly diminishing the effectiveness of surgical and local interventions^2^. Sorafenib, a tyrosine kinase inhibitor, was the first systemic therapy to receive FDA approval for the treatment of advanced, unresectable HCC^3,4^. It effectively prolongs median survival for HCC patients by inhibiting enzymes critical for tumor growth and proliferation, while also exerting anti-angiogenic effects^2,5^. However, the emergence of acquired resistance to sorafenib limits the long-term benefits of systemic treatment for a considerable number of advanced HCC patients^6,7^. The underlying mechanisms of this resistance are complex and multifactorial^5^, involving autophagy regulation^8,9^, abnormal expression of miRNA^10^,metabolic alterations^11^, and remodeling of the tumor microenvironment^12^. While these mechanisms provide valuable insights, the specific processes and key regulators driving sorafenib resistance in HCC remain poorly defined. Therefore, gaining a deeper insight into the mechanisms that lead to resistance, along with developing preventive strategies, is crucial for enhancing treatment outcomes in advanced HCC.

The Hedgehog (HH) signaling pathway, initially identified in Drosophila, is essential for embryonic development and plays a key role in maintaining homeostasis in adult tissues^13^. Research has demonstrated that the HH pathway is involved in the regulation of cancer stem cells (CSCs) and contributes to drug resistance^14,15^. Consequently, abnormal activation of the HH pathway has been shown to facilitate tumor progression and impact the efficacy of clinical treatments across various cancers, including HCC^16,17^, establishing it as a prominent target in cancer therapy research^13,18^. Given that the HH pathway is closely related to the progression and drug resistance of HCC, elucidating the key factors that regulate the HH pathway is crucial for developing strategies to target HCC drug resistance. Tumor heterogeneity is a significant factor contributing to tumor stemness and drug resistance^19,20^. Single-cell RNA sequencing (scRNA-seq) technology offers essential insights into the transcriptomic heterogeneity of relevant cell types^21^, enabling the analysis of cellular subpopulations and their interactions, as well as the study of tumor heterogeneity at the single-cell level^22,23^. Recent studies have highlighted the various heterogeneities of triple-negative breast cancer (TNBC) cells in response to JQ1 treatment, along with the development of resistance, leading to the identification of promising combination therapies aimed at effectively addressing chemotherapy-resistant TNBC^24^. Similarly, Wu et al. demonstrated that IL-1β-driven infiltration of myeloid-derived suppressor cells (MDSCs) and resultant dysfunction of CD8 T cells contribute to heightened resistance to anti-PD-1 therapy in MSI-H/dMMR colorectal cancer^25^. Thus, scRNA-seq empowers the personalization of cancer treatment strategies based on the distinct genomic characteristics of individual cells, offering unprecedented opportunities for cancer diagnosis and the identification of subpopulations responsive to specific drugs^21,26^.

In this study, we focused on the HH signaling pathway and conducted a comprehensive bioinformatics analysis integrating scRNA-seq with extensive RNA-seq data. We identified SLC25A39 as a potential key regulatory factor and validated its predictive capacity through both internal and external cohorts. Our in vitro and in vivo experiments further confirmed that SLC25A39 facilitates the progression of HCC and impacts resistance to sorafenib. We also observed that knocking down SLC25A39 can significantly enhance the inhibitory effect of sorafenib on tumor growth.

In summary, our results suggest that SLC25A39, as a potential key factor in the HH pathway, plays a crucial role in promoting HCC progression as well as enhancing sorafenib resistance. The targeted suppression of SLC25A39 offers a promising and effective strategy to overcome sorafenib resistance. This approach holds promise for improving treatment outcomes and offers new avenues for the management of HCC.

## Results

### Identification of SLC25A39 as a candidate regulator of the hedgehog pathway based on scRNA-seq and bulk RNA-seq screening

**Fig. 1:**
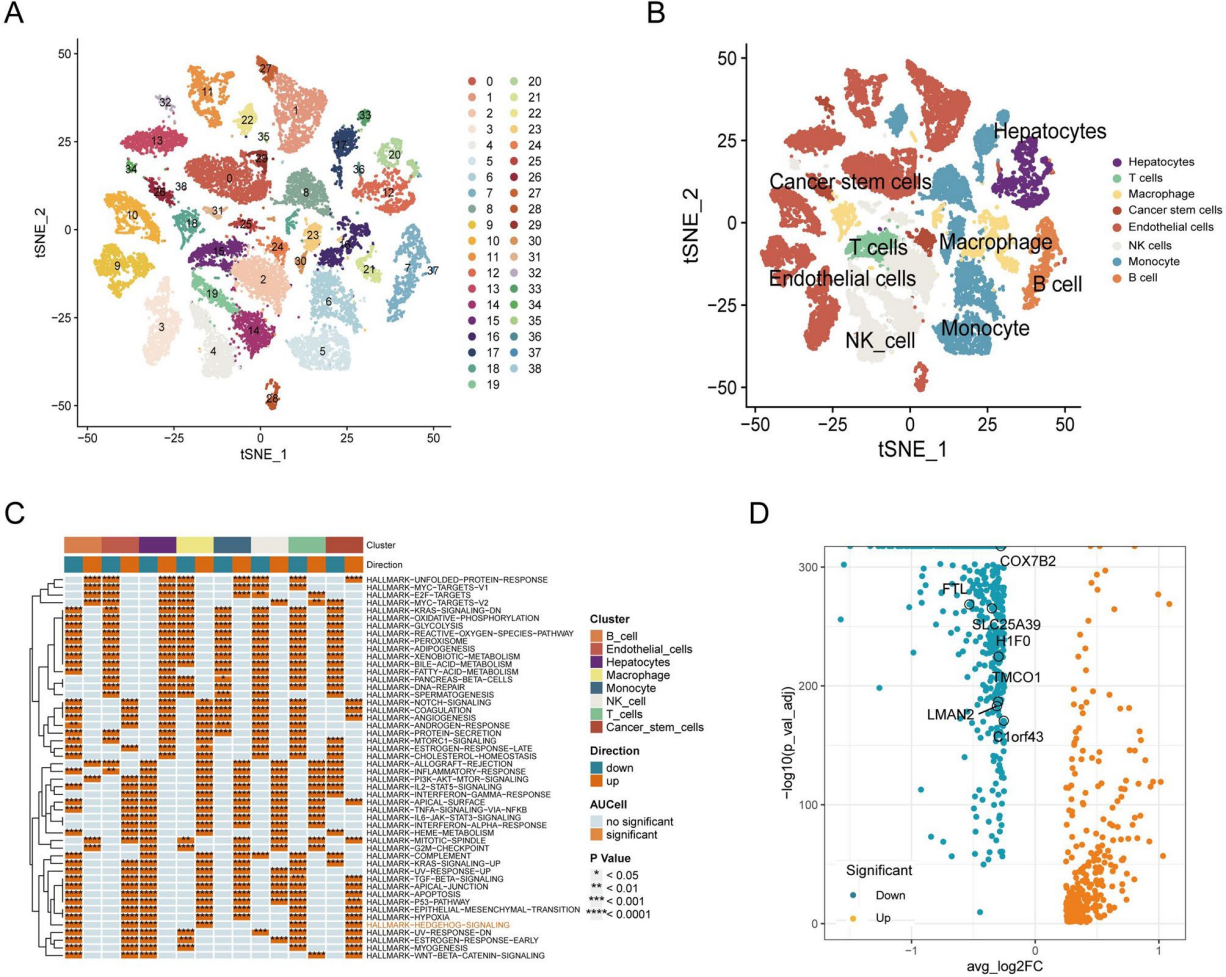
Cell Annotation in HCC Patient Samples and Identification of Hedgehog pathway differential genes. (**A**) t-SNE clustering analysis revealed 39 distinct clusters within the single-cell dataset. (**B**) Cell annotation using the SingleR package classified the cells into eight groups. (**C**) A heatmap illustrating the major enriched signaling pathways across various cell -types. (**D**) Volcano plot showing differential analysis between the high and low Hedgehog pathway score groups across all cell types.

We obtained scRNA-seq data from HCC tissues through the GEO database (GSE149614). Following rigorous quality control and filtering, a total of 10 HCC samples were selected for further analysis. Clustering analysis successfully categorized the samples into 38 distinct clusters (Fig. 1A), with a comprehensive display of the expression profiles of differentially expressed genes within each cluster (Fig. S1A). A comprehensive annotation of these clusters identified eight primary cell types: hepatocytes, T cells, monocytes, endothelial cells, CSCs, natural killer (NK) cells, macrophages and B cells (Fig. 1B). To visualize the key differential gene expressions among these cell types, we generated heatmap (Fig. S1B). Additionally, bubble plots and violin plots displayed the expression of marker genes within each cell type (Fig. S1C-D). The key differential genes identified include ALB in hepatocytes, TRAC in T cells, HLA-DPA1 in monocytes, FABP4 in endothelial cells, C1QB in macrophages, ACTA2 in CSCs, HBB in NK cells, and IGHG3 in B cells.

To further investigate the mechanisms underlying intercellular interactions, we employed CellChat to assess the strength and frequency of interactions among the eight major cell subpopulations. The analysis revealed a significantly enhanced exchange of ligand-receptor signaling between HCC cells and both endothelial cells and macrophages (Fig. S2A). Pathway-level analysis indicated a significant enrichment of the MIF signaling pathway (CD74-CXCR4/CD44 receptor pair) in cross-cell communication (Fig. S2B). Moreover, we delineated the specific pathways activated within each cell subpopulation, with the HH signaling pathway showing significant activation across multiple cell types (Fig. 1C). We also quantified the number of enriched pathways in different cell types (Fig. S2C) and assessed the degree of HH pathway enrichment among various cell populations (Fig. S2D). Finally, using pathway activity scores generated by the AUCell algorithm, we classified cells into high-expression and low-expression groups (Fig. S2E). We then performed differential gene analysis on the high-activity and low-activity groups across all cell types, successfully identifying HH pathway-associated differentially expressed genes (referred to as HH DEGs) (Fig. 1D).

**Fig. 2:**
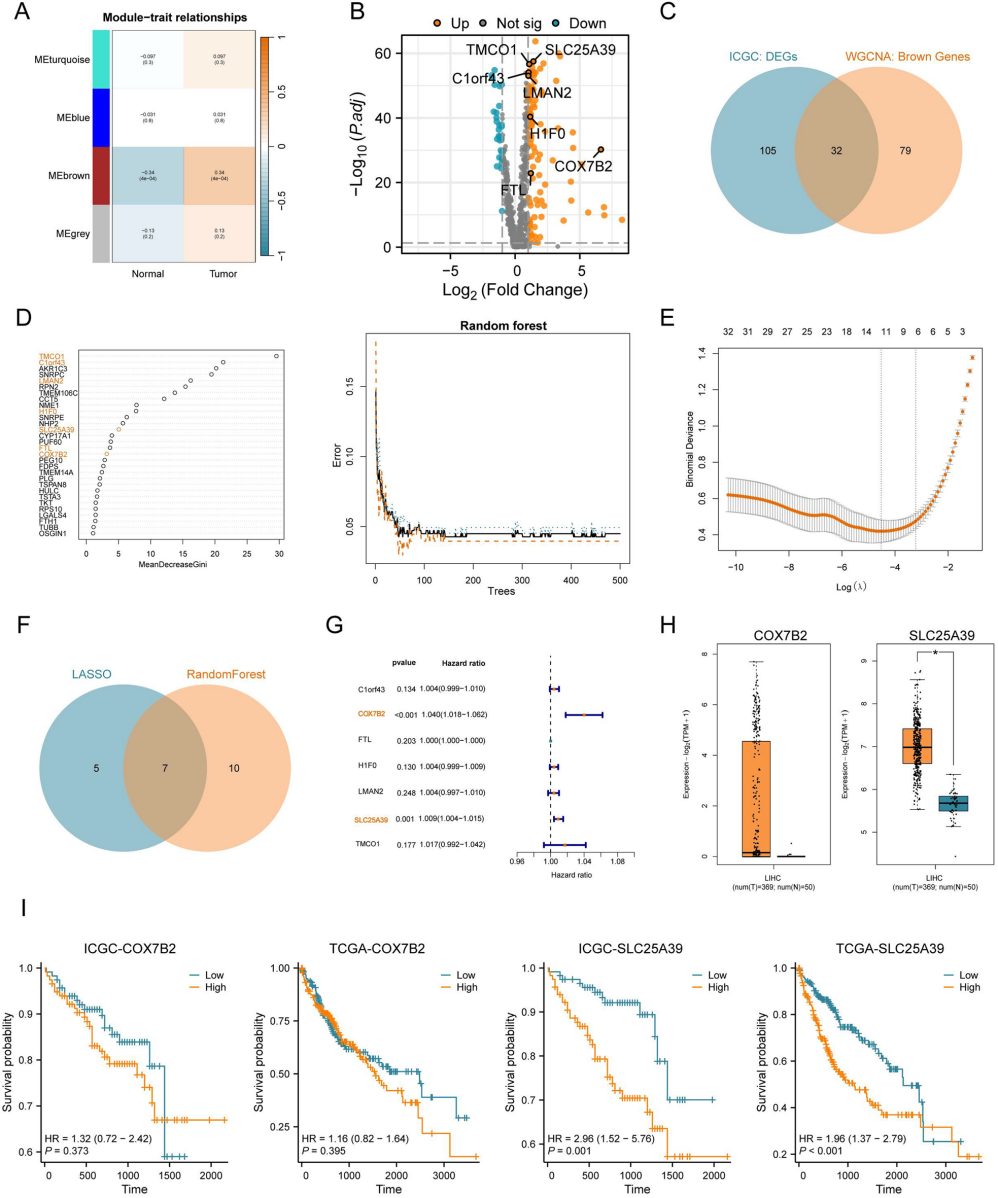
Multidimensional Filtering and Selection of Hedgehog pathway differential genes. (**A**, **B**) Weighted gene co-expression network analysis (WGCNA) and differential analysis of Hedgehog pathway differential genes (HH-DEGs) in the ICGC cohort. (**C**) Venn diagram illustrating the results from WGCNA and differential analysis. (**D**, **E**) Random forest and Lasso regression analyses performed on the filtered genes. (**F**) Venn diagram showing the results from the random forest and Lasso regression analyses. (**G**) A univariate Cox regression analysis was performed on the seven H-DEGs using data from the ICGC database. (**H**) Expression levels of COX7B2 and SLC25A39 in the TCGA-HCC cohort. (**I**) Prognostic assessment of SLC25A39 and COX7B2 from HCC cohorts in TCGA and ICGC. Mean ± SEM. *p < 0.05, **p < 0.01, ***p < 0.001.

Following the identification of HH pathway differential genes (HH-DEGs), we obtained the corresponding HCC bulk RNA-seq data from the International Cancer Genome Consortium (ICGC) cohort. Initially, we performed preliminary filtering using weighted gene co-expression network analysis (WGCNA) and differential analysis (Fig. 2A-C). Following this, we employed two machine learning algorithms—random forest and LASSO regression—for further filtering and selection (Fig. 2D, E), ultimately identifying seven core genes(Fig. 2F). To assess the clinical relevance of these core genes, we integrated survival data from ICGC-HCC patients and performed univariate Cox regression analysis on the identified gene set (Fig. 2G), This analysis highlighted SLC25A39 and COX7B2 as key genes of interest. However, unlike SLC25A39, COX7B2 expression showed no significant difference in the TCGA-HCC cohort (Fig. 2H). Additionally, prognostic analyses conducted using HCC cohorts from both TCGA (https://www.cancer.gov/ccg/) and ICGC (https://dcc.icgc.org/) datasets confirmed that COX7B2 does not possess predictive value for patient outcomes (Fig. 2I)(Original data see Supplementary Information 2). We also further validated our findings through Cox regression analyses within the TCGA cohort, which demonstrated that SLC25A39 maintains a more consistent hazard ratio (Fig. S2F). Moreover, in dataset GSE189903, SLC25A39 was consistently identified as a differentially expressed gene associated with the HH pathway, whereas COX7B2 was not detected difference (Supplementary Information 3). Based on this comprehensive evaluation, we ultimately designated SLC25A39 as a promising therapeutic target in hepatocellular carcinoma.

### Upreguation of SLC25A39 in HCC and its association with poor prognosis

**Fig. 3:**
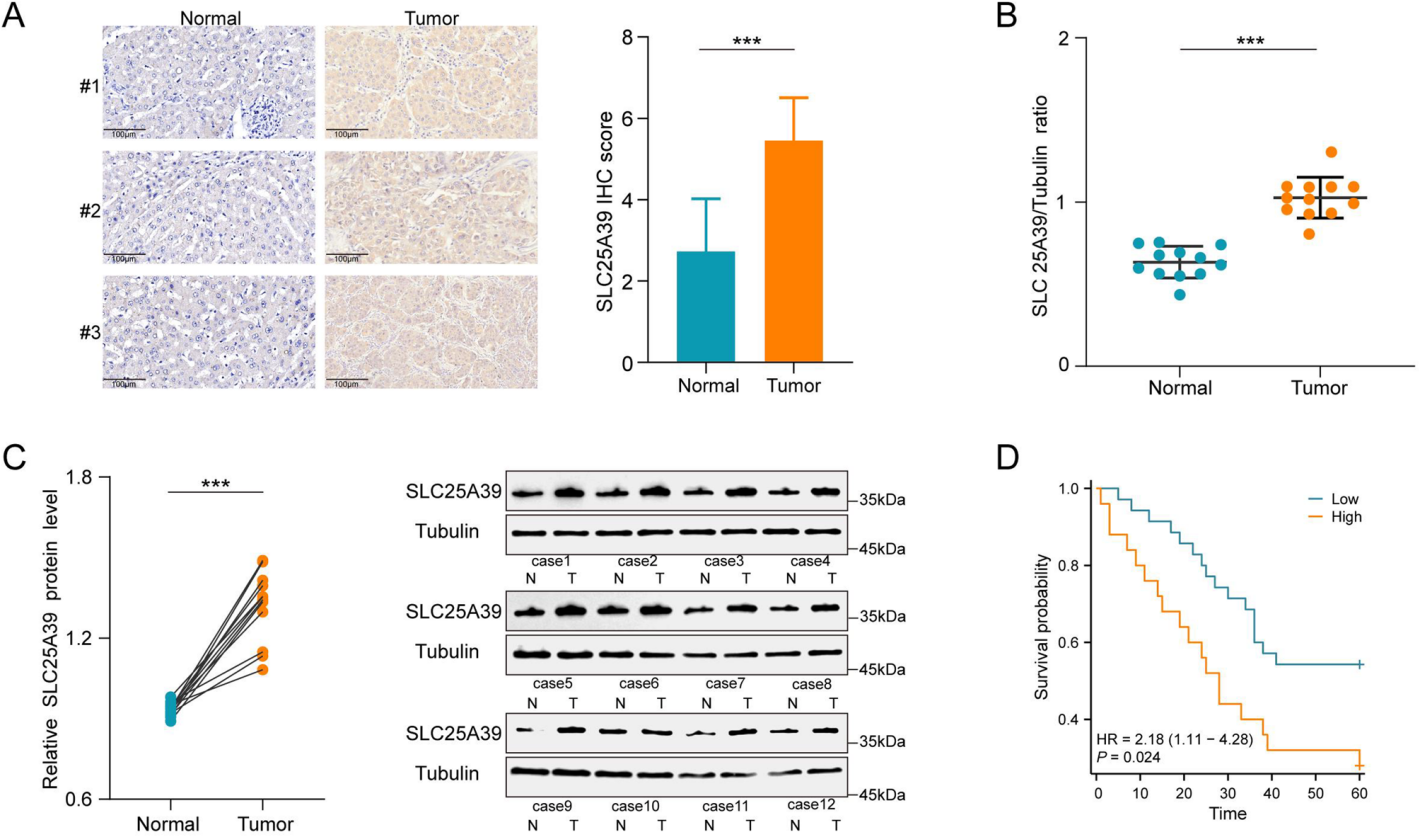
Upregulation of SLC25A39 in HCC Patients Correlates with Poor Prognosis. (**A**) IHC assessment of SLC25A39 expression in samples from HCC patients and adjacent non-tumor tissues (n = 60). Scale bars, 100 μm. (**B**) qRT-PCR analyses of SLC25A39 mRNA expression levels in 12 pairs of HCC and adjacent non-tumor tissues. (**C**) Western blot analysesof SLC25A39 protein levels in 12 pairs of HCC and adjacent non-tumor tissues. (**D**) Overall survival (OS) constructed based on data from HCC patients(n = 60). Mean ± SEM. *p < 0.05, **p < 0.01, ***p < 0.001.

To explore the clinical relevance of SLC25A39 in HCC, we conducted a study on its expression and prognostic value. Initially, immunohistochemical staining on paraffin-embedded sections from 60 HCC patients indicated that the expression of SLC25A39 was elevated in tumor tissues compared to adjacent tissues (Fig. 3A). This finding was further supported by additional qRT-PCR (Fig. 3B) and Western blot analyses (Fig. 3C) conducted on fresh tumor and adjacent tissues from 12 HCC patients. Prognostic analysis based on immunohistochemical staining scores demonstrated that higher SLC25A39 expression is associated with shorter overall survival (OS) (Fig. 3D), which is consistent with our previous findings. Furthermore, elevated expression of SLC25A39 is significantly associated with tumor differentiation and median survival time, while exhibiting no correlation with pathological characteristics such as sex, age, or vascular invasion (Table 1). Overall, our results indicate that SLC25A39 is instrumental in regulating disease progression and prognosis in HCC patients, highlighting its potential as a novel therapeutic target.

**Table 1:** Distribution of Clinicopathological Characteristics According to Expression Levels.

Clinicopathological characteristics	Low-expression	High-expression	P-value
**Age(years)**			
≥ 60	15	11	0.9298
< 60	20	14	
**Sex**			
Male	26	21	0.5275
Female	9	4	
**Stage**			
I + II	33	21	0.2234
III + IV	2	4	
**HBV**			
Postive	20	15	0.8248
Negtive	15	10	
**Vascular invasion**			
Yes	10	6	0.693
No	25	19	
**Tumor differentiation**			
Poorly	3	8	0.0392*
Well	32	17	
Longest diameter of tumor(mm)	44.06	51.32	0.4067
AST	42.35	112.7	0.0945
ALT	44.62	68.22	0.0633
Median OS	43.26	31.92	0.0372*

### Knockdown of SLC25A39 inhibits HCC progression

**Fig. 4:**
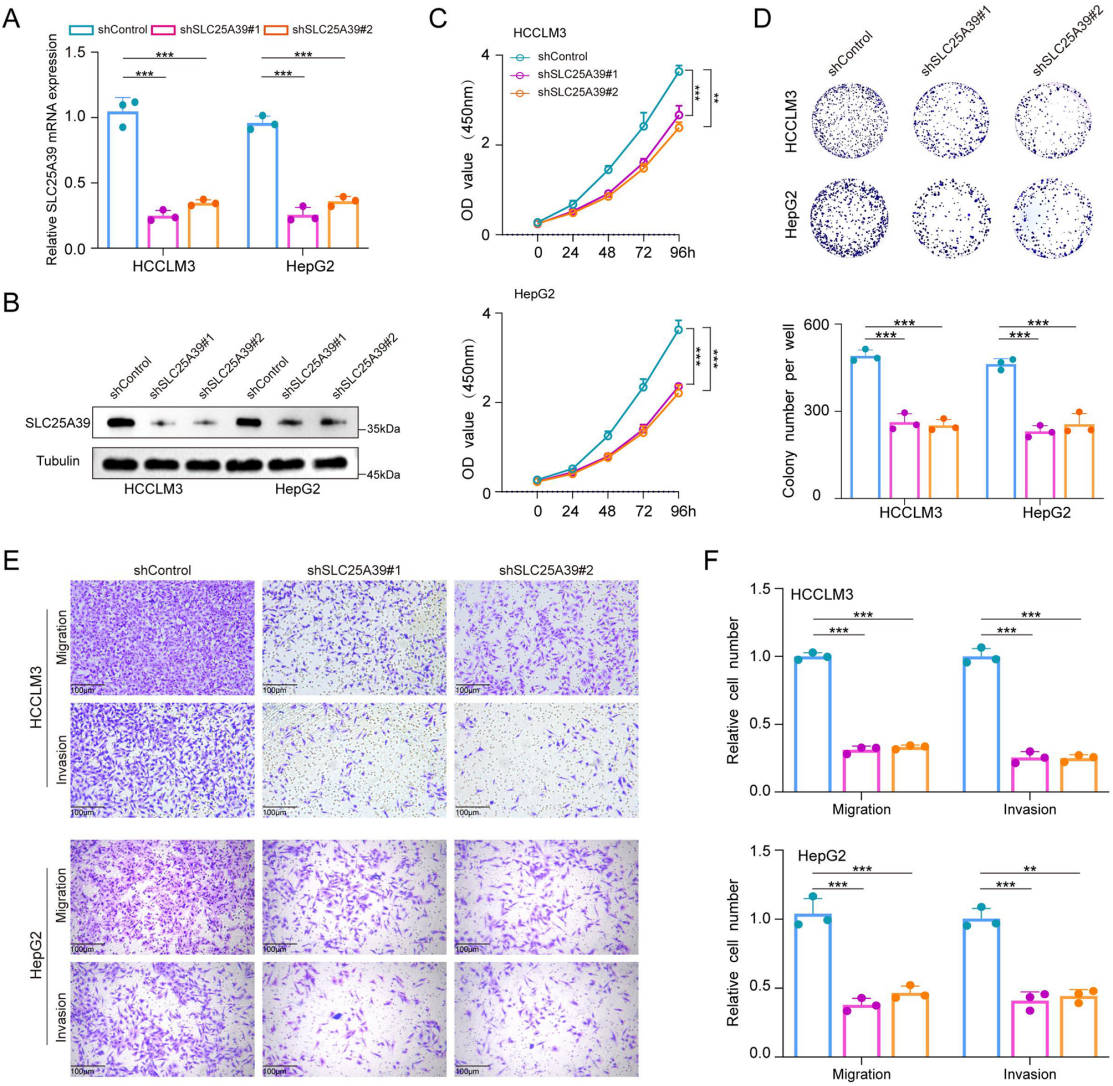
Knockdown of SLC25A39 Inhibits Proliferation and Migration of HCC Cells. (**A**, **B**) Western blot and qRT-PCR analyses of transfection efficiency for shSLC25A39 and shControl in HCCLM3 and HepG2 cells. (**C**, **D**) CCK-8 and colony formation assays assessing the impact of shSLC25A39 or negative control on cell proliferation. (**E**, **F**) Transwell assays to evaluate the migration and invasion potential of shSLC25A39 and negative control cells.Scale bars, 100 μm. Mean ± SEM. *p < 0.05, **p < 0.01, ***p < 0.001.

To assess the role of SLC25A39 in the progression of HCC, we initially examined its expression levels in HCC cell lines (Fig. S3A, B). Based on the expression of SLC25A39, we subsequently established stable knockdown HCC cell lines in HCCLM3 and HEPG2 cells and validated the knockdown efficiency using Western blotting and qRT-PCR (Fig. 4A, B). To investigate the effects of SLC25A39 knockdown on cell proliferation, we conducted CCK-8 assays (Fig. 4C) and colony formation assays (Fig. 4D). The results indicated that knockdown of SLC25A39 significantly weakened the proliferation abilities of HCCLM3 and HEPG2 cells. Additionally, we used Transwell assays to assess the impact of SLC25A39 knockdown on cell migration and invasion. Our findings revealed that shSLC25A39 significantly inhibited the migration and invasion capabilities of HCCLM3 and HEPG2 cells compared to controls (Fig. 4E, F). Overall, these results suggest that SLC25A39 is crucial for the proliferation, migration, and invasion of HCC cells, highlighting its significance in disease progression.

### Depletion of SLC25A39 weakens CSCs properties and sorafenib resistance in HCC

**Fig. 5:**
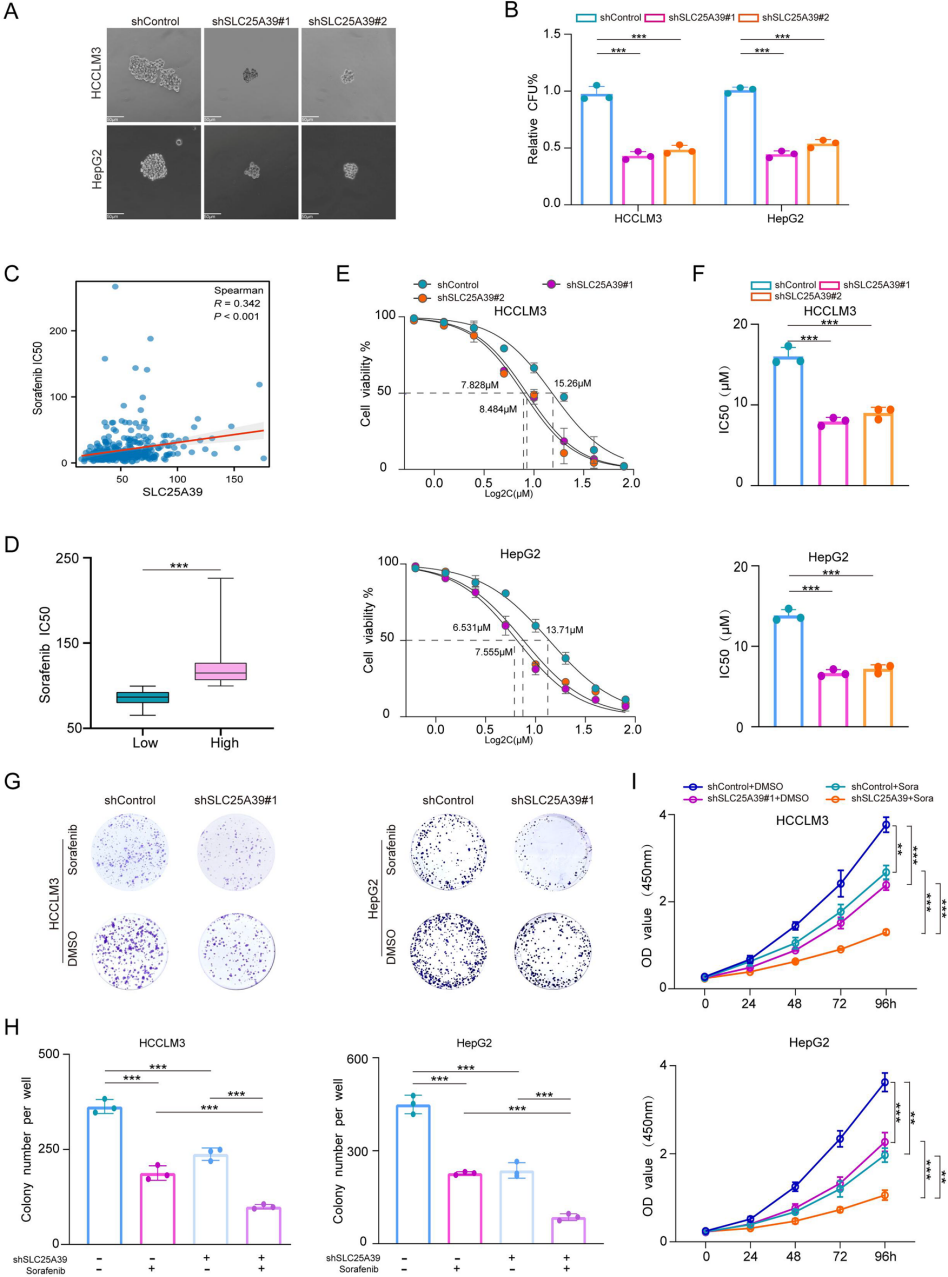
SLC25A39 Deficiency Suppresses Stemness Characteristics and Drug Resistance in HCC Cells. (**A**, **B**) Tumorsphere assays conducted on HCC cells transfected with shSLC25A39. Scale bars, 50 μm. (**C**, **D**) Analysis of SLC25A39 expression in relation to sorafenib sensitivity in HCC patients. (**E**, **F**) Variations in sorafenib IC50 values for HCCLM3 and HepG2 cells following stable knockdown of SLC25A39 compared to control. (**G**, **H**) Colony formation assays performed with plasmid-transfected cells in the presence of sorafenib. (**I**) CCK-8 assays conducted with plasmid-transfected cells in the presence of sorafenib. Mean ± SEM. *p < 0.05, **p < 0.01, ***p < 0.001.

To further validate the potential correlation between SLC25A39 and the HH signaling pathway, we initially performed qRT-PCR analysis on some key target genes of the HH pathway: Gli1,, Smo, Gli3, and Ptch1. The results showed that downregulation of SLC25A39 led to decreased expression of Gli1,and Smo, while increased expression of Gli3 and Ptch1 (Fig. S4A). The HH signaling pathway is known to play a crucial role in regulating cell differentiation and the development of drug resistance^14,15^.This observation leads us to propose a bold hypothesis: that abnormal expression of SLC25A39 may influence the stemness characteristics and drug resistance in HCC. To explore this hypothesis, we conducted sphere formation assays, a classical method for assessing cellular stemness^27^. The results revealed that knockdown of SLC25A39 significantly reduced the ability to form spheroids (Fig. 5A, B). Furthermore, the shSLC25A39 group demonstrated a marked decrease in the expression of surface markers associated with CSCs (Fig. S4B).

Meanwhile, we also explored the impact of SLC25A39 on sorafenib resistance. Based on our previous study^28^, we used the “oncoPredict” package^29^ to evaluate the drug sensitivity of TCGA-HCC patients to sorafenib.This analysis revealed a significant positive correlation between SLC25A39 expression levels and sorafenib IC50 values (Fig. 5C) indicating that patients with higher levels of SLC25A39 exhibited poorer sensitivity to the drug (Fig. 5D). Additionally, as shown in Figure S3C, shRNA-mediated depletion of SLC25A39 led to significant downregulation of various ABC transport proteins, including ABCB1, ABCC1, and ABCG2^30,31^. We further evaluated the IC50 values of cells treated with varying concentrations of sorafenib using the CCK-8 assay, our results demonstrated that the knockdown of SLC25A39 significantly enhanced the sensitivity of HCC cells to sorafenib (Fig. 5E, F). Additionally, colony formation(Fig. 5G, H) and CCK-8 (Fig. 5I) assays reinforced that silencing SLC25A39 increased sorafenib sensitivity in HCC cells.Overall, these results underscore the critical role of SLC25A39 in maintaining CSCs properties and contributing to sorafenib resistance in HCC.

### ShSLC25A39 synergistically enhances sorafenib-induced apoptosis in tumor cells, inhibiting tumor growth

**Fig. 6:**
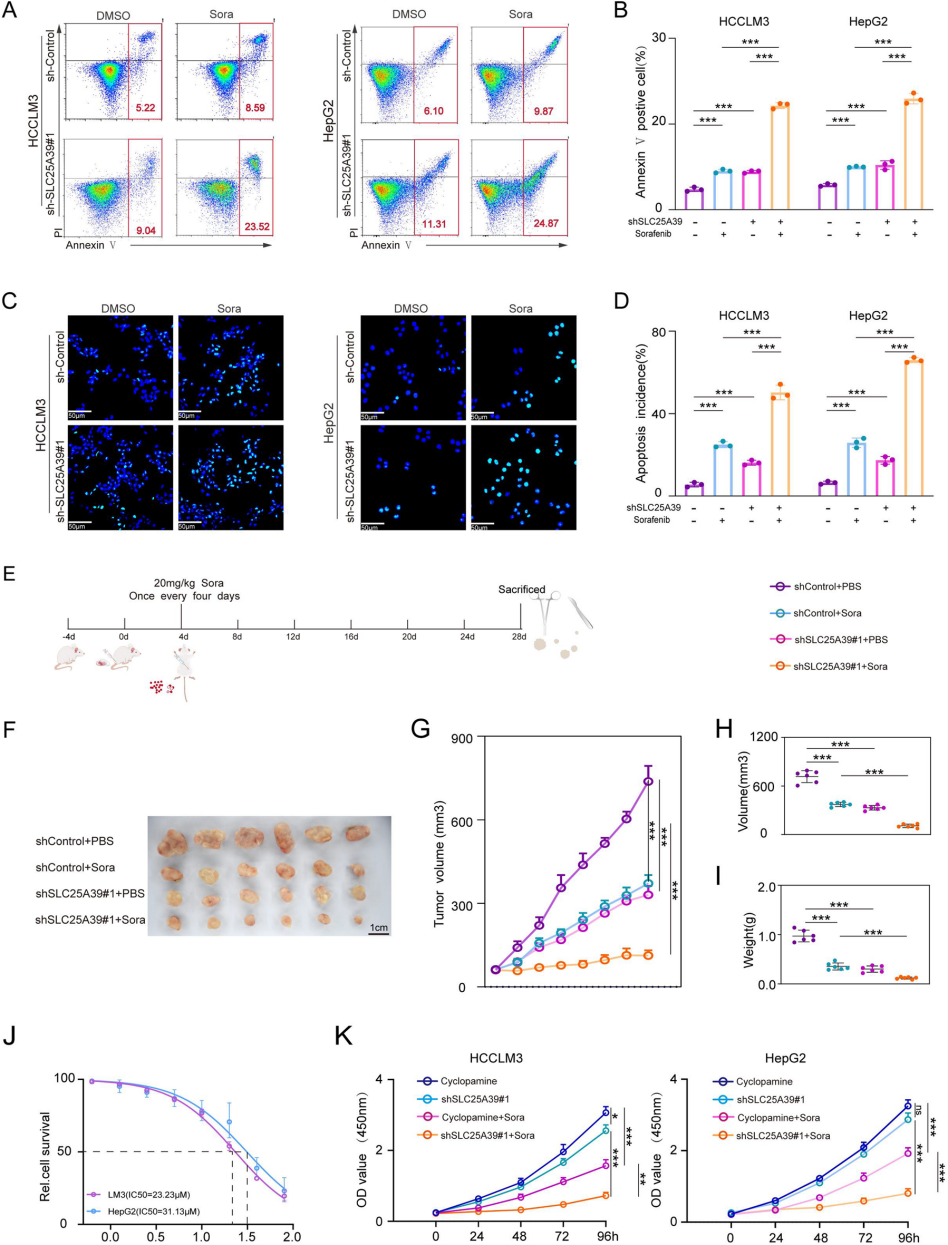
ShSLC25A39 Synergistically Enhances the Inhibitory Effects of Sorafenib on Tumor Growth. (**A**, **B**) Flow cytometric analysis of apoptosis was performed using FITC and PI staining in cells treated with or without sorafenib and shSLC25A39. (**C**, **D**) TUNEL staining analysis was conducted to assess apoptosis in cells treated with or without sorafenib and shSLC25A39.Scale bars, 50 μm. (**E**) A schematic diagram illustrating the subcutaneous tumor formation model: stable-transfected HCC cells were injected into the right inguinal region of nude mice. Tumor size was measured every four days. Concurrently, the mice received intraperitoneal injections of sorafenib (20 mg/kg). On day 28, the mice were euthanized, and the subcutaneous tumors were excised and weighed. n = 6 mice/group. (**F**, **G**) Quantification of tumor growth across different treatment groups. (**H**, **I**) Comparisons of tumor size and weight among the various treatment groups. (**J**) Cyclopamine IC50 values for HCCLM3 and HepG2 cells. (**K**) CCK-8 assays were performed to evaluate cell viability following treatment with sorafenib combined with shSLC25A39 or cyclopamine. Mean ± SEM. *p < 0.05, **p < 0.01, ***p < 0.001.

Previous studies have suggested that SLC25A39 plays a critical role in maintaining stemness characteristics and conferring resistance to sorafenib. To further elucidate the therapeutic effect of the combination of SLC25A39 and sorafenib, we first conducted membrane protein V and propidium iodide (PI) staining (Fig. 6A, B), along with TUNEL assays (Fig. 6C,D). These analyses indicated that both sorafenib alone and SLC25A39 knockdown promoted apoptosis, with a synergistic effect observed when these treatments were combined. Consistent results were obtained from qRT-PCR analysis of apoptosis-related factors (Fig. S5A).

Next, we assessed the in vivo efficacy of these treatments through subcutaneous tumor implantation experiments in nude mice. The mice were randomly assigned to four groups: shControl + PBS, shSLC25A39 + PBS, shControl + Sorafenib, and shSLC25A39 + Sorafenib. Drug treatments were administered as illustrated in the schematic diagram (Fig. 6E). The results showed that knockdown of SLC25A39 significantly inhibited tumor growth compared to the control group, and further combination with sorafenib could enhance the effect of inhibiting tumor growth (Fig. 6F-I).

To further assess the therapeutic potential of combining SLC25A39 knockdown with sorafenib, we first determined the IC50 of cyclopamine (MCE, HY-17024), a specific HH pathway inhibitor, to identify an appropriate drug concentration (Fig. 6J). Subsequently, cell viability was evaluated using the CCK-8 assay across four treatment groups: cyclopamine alone, cyclopamine combined with sorafenib, shSLC25A39 alone, and shSLC25A39 combined with sorafenib. Our results consistently showed that the combination treatments exerted a significantly greater inhibitory effect on cell viability compared to either agent alone. Notably, the combination of SLC25A39 knockdown with sorafenib produced the most substantial reduction in cell viability, indicating a potential synergistic effect that may enhance therapeutic efficacy against hepatocellular carcinoma (Fig. 6K).These results suggest that SLC25A39 may function as an upstream regulator, and its inhibition could offer broader therapeutic benefits. Meanwhile, We also observed that the combined treatment of shSLC25A39 and sorafenib appeared to partially influence HH pathway markers, although the effects were not complete(Fig. S5B). This suggests that HH signaling might be involved to some extent. Howevwe, the detailed molecular mechanism still needs further exploration in the future.

## Discussion

Sorafenib has demonstrated significant antitumor efficacy in advanced HCC patients; however, the emergence of drug resistance is quite common. This resistance is often linked to the overexpression of ATP-binding cassette (ABC) transporters, which enhances drug efflux and can result from mutations in drug targets, cancer cell adaptation to the tumor immune microenvironment, and changes in the hydrophobicity of effluxed drugs^32–35^.Key members of this group that have been extensively studied include ABCB1, ABCG2, and ABCC1^36^. In addition to these overexpressed transport proteins, the WNT, Notch, and HH pathways are also critical in the development of drug resistance^37^.Notably, the abnormal activation of the HH pathway has garnered significant attention for its contribution to cancer cell stemness and resistance^38^,particularly in the context of sorafenib resistance. Several reports indicate that alterations in HH signaling can significantly impact clinical outcomes^39,40^. However, there is currently a lack of systematic evaluations of key regulators in the HH pathway.

This study aims to integrate bioinformatics and fundamental biological strategies to investigate the HH pathway and its role in HCC, thereby exploring key targets that drive HCC progression and sorafenib resistance. In this research, we first performed a systematic bioinformatics analysis using scRNA-seq and bulk RNA-seq data from HCC patients and found that the solute carrier family member SLC25A39 may play an important role in the HH pathway of HCC. Interestingly, recent study suggest that SLC transporters, similar to ABC proteins, are involved in the transport of various solutes across biological membranes, which has significant clinical implications for drug absorption, metabolism, distribution, and excretion^41,42^.Changes in SLC transporter activity can lead to pharmacokinetic alterations, ultimately affecting drug efficacy^43,44^. For example, in KRAS-mutant colorectal cancer cells, downregulation of SLC25A22 inhibits DNA demethylation, enhancing WNT signaling and promoting stemness and drug resistance^45^. Furthermore, studies on resistance to doxorubicin and epirubicin have shown that downregulation of SLC38A7 and SLC46A1 can lead to decreased drug sensitivity^46^. These findings underscore the potential importance of SLC25A39 in cancer progression and drug resistance.

Notably, previous studies have identified SLC25A39 as a key member of the solute carrier (SLC) transporter family, playing an essential role in iron homeostasis and the mitochondrial transport of glutathione (GSH)^47–49^. These functions are consistent with our clinical findings, as our internal data analysis revealed a trend toward elevated serum AST and ALT levels in the high SLC25A39 expression group. Although these differences did not reach statistical significance—likely due to limited sample size—we suspect that a broader cohort may confirm a potential association. This finding suggests a possible link between SLC25A39 and liver injury, particularly mitochondrial stress. Given the established relationship between GSH metabolism, mitochondrial oxidative stress, and sorafenib resistance in HCC^50,51^, We hypothesize that SLC25A39 may mediate the activation of HH pathway by regulating mitochondrial iron homeostasis and GSH levels, thereby influencing sorafenib-associated hepatotoxicity and metabolic adaptation. Further mechanistic investigations are critical to elucidate how SLC25A39 mediates drug resistance and liver injury, as such insights could reveal novel therapeutic targets for overcoming sorafenib resistance in HCC. Overall, these findings underscore the potential importance of SLC25A39 in cancer progression and therapeutic resistance, warranting continued exploration of its biological functions and clinical implications.

This study found that elevated expression levels of SLC25A39 are linked to poor prognosis in HCC patients and reduced responsiveness to sorafenib. Functionally, our findings reveal that knockdown of SLC25A39 expression significantly inhibited HCC progression and diminishes stemness characteristics. Additionally, in vitro and in vivo experiments confirmed that the combination of SLC25A39 knockdown with sorafenib treatment further inhibited cancer progression. These observations align with our functional experimental results, indicating that abnormally high expression of SLC25A39 not only promotes HCC progression but also aggravates sorafenib resistance.

Like similar studies, our study also has some limitations. On the one hand, we emphasized but did not fully analyze the specific molecular mechanism by which SLC25A39 regulates the HH pathway to affect sorafenib resistance in HCC. In particular, SLC25A39, as a mitochondrial glutathione transporter, has not yet been clearly defined as the cascade reaction that mediates HH pathway activation through iron homeostasis or oxidative stress. Secondly, our animal experiments relied on a subcutaneous xenograft model, which does not fully mimic the tumor microenvironment of in situ HCC, including immune microenvironment influences on drug resistance. In future studies, we will focus on analyzing the mechanism of action of SLC25A39 in affecting sorafenib resistance in HCC, and comprehensively analyze the drug resistance regulatory function of SLC25A39 in the tumor microenvironment by optimizing the HCC animal model. Ultimately, large scale clinical validation is crucial for ensuring the reliability of our conclusions. Future efforts should focus on broader validation to strengthen and extend our findings.

In summary, this study reveals, for the first time, the potential mechanism by which SLC25A39 regulates stemness and sorafenib resistance in HCC. This discovery not only deepens our understanding of the mechanisms underlying stemness and drug resistance in HCC but also presents promising avenues for future prognostic assessments and combined targeted therapies aimed at overcoming sorafenib resistance. We anticipate that future research will further validate these findings and explore effective strategies to leverage these mechanisms to improve treatment outcomes for HCC patients.

## Materials and methods

### Patient information for HCC

We collected 60 paraffin-embedded specimens from HCC patients treated at the First Affiliated Hospital of Anhui Medical University. The clinical data utilized in this study were approved by the hospital’s Ethics Committee(LLSC2020979), and informed consent was obtained from all participants.

### Data collection

Single-cell datasets for HCC were retrieved from the GEO database (GSE149614, GSE189903). Bulk RNA sequencing data and prognostic information were sourced from the ICGC database (https://dcc.icgc.org/), while differential expression and prognostic information regarding SLC25A39 in HCC patients were sourced from the GEPIA2 database (http://gepia2.cancer-pku.cn/).

### Single-cell data analysis

The single-cell RNA sequencing data were processed using the R package “Seurat” (v4.3.0). Initial quality control was stringent, retaining cells that expressed more than 50 genes (nFeature_RNA > 50) and displayed less than 5% mitochondrial gene contribution (percent_MT < 5). After normalization and principal component analysis (PCA), cells were clustered into distinct groups using the FindClusters function with a resolution parameter of 0.8. The SingleR package was utilized for cellular type identification, categorizing the cells into eight major types: hepatocytes, T cells, monocytes, endothelial cells, macrophages, CSCs, natural killer (NK) cells, and B cells.

### Cell communication analysis

To examine intercellular communication networks, we employed the “CellChat” package. Using the quality-controlled and annotated single-cell dataset, we constructed a “CellChat” object following the recommended protocols. By integrating ligand-receptor interaction information from the CellChatDB database, we calculated the specific communication signal strength between cell subpopulations, with statistical significance determined at p < 0.05.

### Cell pathway activity scoring

HH pathway-associated gene sets were downloaded from the MSigDB database, and pathway activity scores (Hedgehog Score) were computed for each cell based on gene expression rank arrays using the AUCell algorithm. Cells were categorized into high-activity (Hedgehog high) and low-activity (Hedgehog low) groups based on median scores. Differential gene analysis was performed using the Wilcoxon rank-sum test, with selection criteria set at |log2FC|> 1 and p < 0.05.

### Functional enrichment analysis

Functional enrichment scores for pre-defined gene sets were calculated using the irGSEA package. Each cell subpopulation was systematically evaluated for enriched pathways based on annotated cell types. Enrichment analysis utilized hallmark gene sets from MSigDB, defining significant pathways at p < 0.05. Visualization of results was performed using t-SNE dimensionality reduction and differential heatmaps.

### Cell culture

HCC cell lines (MIHA, HCCLM3, Huh7, MHCC97H, MHCC97L, and HepG2) were were purchased from the Cell Bank of Type Culture Collection of the Chinese Academy of Sciences (Shanghai, China), maintained in high-glucose DMEM (Vivacell, Shanghai, China) enriched with 10% fetal bovine serum (Vivacell, Shanghai, China) and 1% penicillin–streptomycin solution (Beyotime Biotechnology, Shanghai, China) at 37 °C in a humidified environment with 5% CO_2_.

### Animal experiments

Six-week-old male nude mice were obtained from Gempharmate (Jiangsu, China) and were kept in specific pathogen-free conditions.Confirms that all experiments were performed in accordance with ethical guidelines approved by the Ethics Committee of the First Affiliated Hospital of Anhui Medical University(LLSC20200965), following NIH standards for animal welfare. The specific experimental process of constructing the subcutaneous tumor model in nude mice was as described in our previous study^52^. In brief, we injected 5 × 10^6 cells of each subgroup into the right inguinal fossa of mice subcutaneously, and recorded the tumor size (including long and short diameters) every 4 days for a total of 7 times. At the same time, in the sorafenib treatment experiment, the experimental mice were injected intraperitoneally with 20 mg/kg sorafenib (MCE, HY-10201) on days 4, 8, 12, 16, 20 and 24, respectively. On day 28, mice were anesthetized using inhaled isoflurane to ensure minimal distress before being euthanized via cervical dislocation. Subcutaneous tumor masses were then removed and weighed.

### Cell transfection

SLC25A39 knockdown in HCCLM3 and HepG2 cells was achieved using specific shRNA sequences (Sangon Biotech, Shanghai, China). Cells were infected with lentivirus for 72 h and selected using puromycin (4 μg/mL, Solarbio). Against SLC25A39 (Sangon Biotech, Shanghai,China) was transfected into the cells using JetPRIME. We verified the knockdown efficiency of shRNA using qRT-PCR and Western Blot. The sequence information and the relevant reagents, including antibodies, are available in Supplementary Information 5.

### Flow cytometry

HCC cells were harvested, washed with phosphate-buffered saline (PBS), and resuspended in binding buffer. Cells were incubated on ice in the dark with Annexin V-FITC (5 μL) for 15 min, followed by the addition of 5 μL of propidium iodide (PI) and an additional 5-min incubation. Samples were analyzed using a BD FACSCelesta (BD Biosciences, USA), and flow cytometry data were processed with NovoExpress software.

### TUNEL staining

Cell slides were fixed with 4% paraformaldehyde and treated with a permeabilization solution (0.1% Triton X-100) to enhance membrane permeability. The TUNEL reaction solution was prepared according to the kit instructions and evenly applied to the samples, followed by incubation at 37 °C for 1 h. After washing with PBS, DAPI solution was added to the samples for 5 min at room temperature for nuclear staining. Mounting medium was applied, and TUNEL staining signals were observed and analyzed using a fluorescent microscope to assess apoptosis levels.

### Immunohistochemistry

Paraformaldehyde-fixed, paraffin-embedded sections from HCC patients were subjected to deparaffinization and rehydration, followed by microwave-assisted antigen retrieval using citrate buffer. Endogenous peroxidase activity was eliminated using hydrogen peroxide, and non-specific binding was minimized through the application of goat serum. The sections were then incubated overnight at 4 °C with a primary antibody targeting SLC25A39 (Bioss, Beijing, China). The following day, enzyme-linked secondary antibodies (Goldbridge Biotechnology, Beijing, China) were applied for 1 h at room temperature. After DAB staining, the sections were washed, counterstained with hematoxylin, dehydrated through an ethanol gradient, clarified in xylene, and finally mounted with neutral resin. Immunohistochemical scores were assigned by two independent professional pathologists.

### CCK-8 assay

Cell viability and IC50 values following treatment were assessed using the CCK-8 assay (Beyotime, Shanghai, China). HCC cells were plated in 96-well plates and allowed to adhere before being subjected to designated treatments. Subsequently, 10 μL of CCK-8 solution was added to each well, and the cells were incubated at 37 °C for 2 h. The absorbance was then measured at 450 nm using a microplate reader.

### Colony formation assay

Treated cells were digested and plated at a density of 1,000 cells per well in 6-well plates, with the culture medium replaced every three days. After 10 days, the experiment was concluded. The cells were subsequently fixed with 4% formaldehyde and stained with 0.1% crystal violet. Cell quantification was performed using ImageJ software.

### Transwell assay

Without or with Matrigel (ABW, Shanghai, China) was added to the upper chamber of the transwell chamber for the assessment of cell migration and invasion. Approximately 5 × 10^4 cells were seeded in the upper chamber containing 250 μL of serum-free medium, while 800 μL of complete medium was added to the lower chamber. After incubating for 24 h, the cells were fixed with 4% formaldehyde and stained with 0.1% crystal violet. Non-migrated cells on the upper surface were carefully removed using a cotton swab, and images were captured with an inverted microscope to quantify the migrated cells.

### Sphere formation assay

HCC cells (1 × 10^3 cells per well) were plated in ultra-low attachment 24-well plates (Corning, USA) and cultured in DMEM/F12 supplemented with 1% B-27 (Invitrogen, USA), 1% N-2 (Invitrogen, USA), 20 ng/mL EGF (Peprotech, USA), and 20 ng/mL bFGF (Peprotech, USA) for one week. After this incubation period, the number of spheres with a diameter exceeding 50 μm was counted under a microscope.

### Quantitative and statistical analysis

All data were analyzed and visualized using GraphPad Prism (version 8.0.2). A p-value of less than 0.05 was considered statistically significant, with the following designations: ns for not significant; * for p < 0.05; ** for p < 0.01; and *** for p < 0.001.

## Supplementary Information

Supplementary Information 1.

Supplementary Information 2.

Supplementary Information 3.

Supplementary Information 4.

Supplementary Information 5.

## Data Availability

The original contributions presented in the study are included in the article/Supplementary Material. Further inquiries can be directed to the corresponding authors.
