# Supplementary figures and images for: SLC25A39 regulates Hedgehog signaling to promote tumor progression and sorafenib resistance in hepatocellular carcinoma
Source: Sci Rep. 2025 Oct 15;15:36061. doi: 10.1038/s41598-025-20008-7 (PMC12528477; doi:10.1038/s41598-025-20008-7)

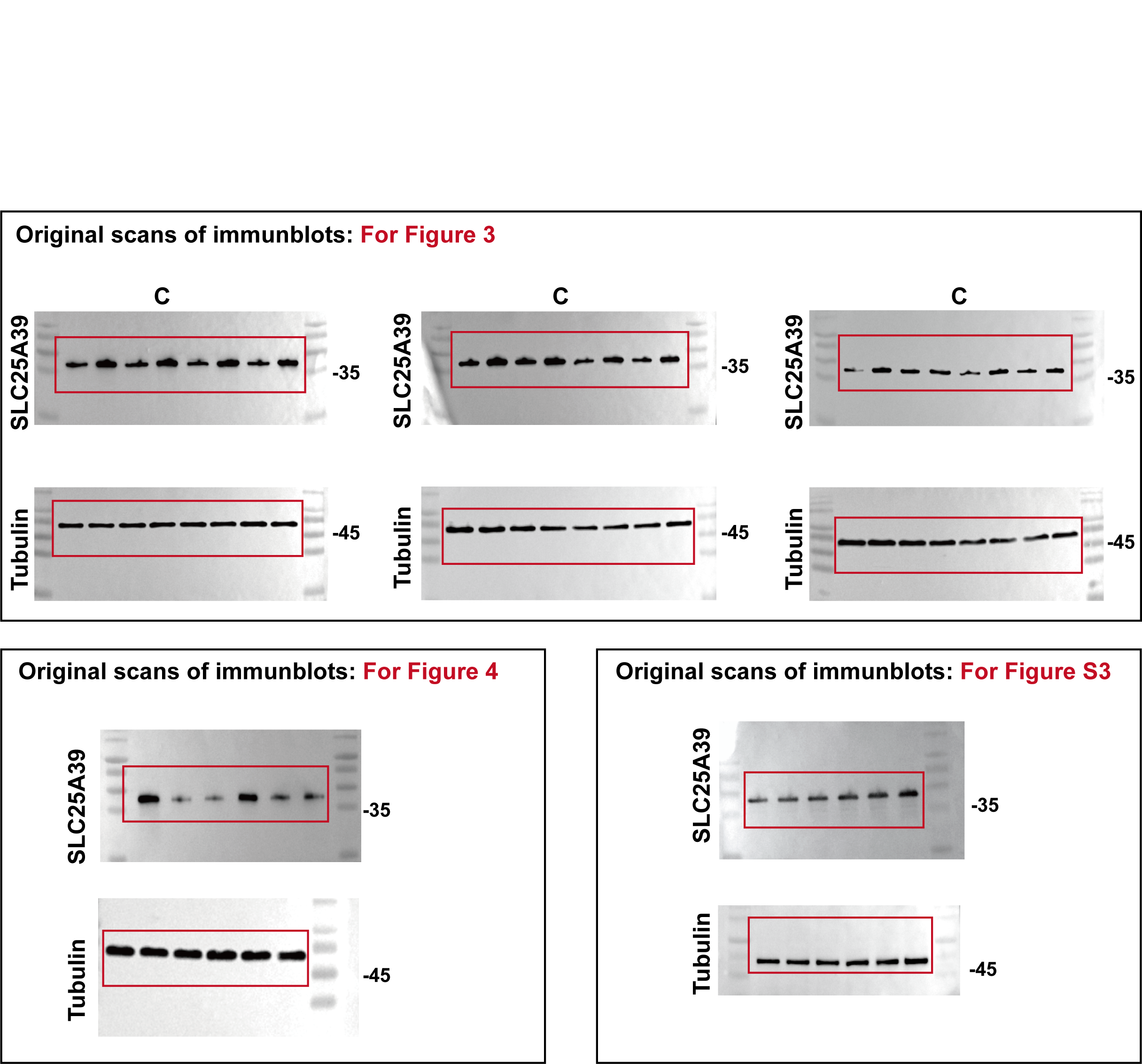

Supplement: Supplementary file 1 — Supplementary Information 1. [file 41598_2025_20008_MOESM1_ESM.tif]
