# Supplementary material for: SLC25A39 regulates Hedgehog signaling to promote tumor progression and sorafenib resistance in hepatocellular carcinoma
Source: Sci Rep. 2025 Oct 15;15:36061. doi: 10.1038/s41598-025-20008-7 (PMC12528477; doi:10.1038/s41598-025-20008-7)
Supplement: Supplementary file 4 — Supplementary Information 4. [file 41598_2025_20008_MOESM4_ESM.pdf]

SLC25A39 Regulates Hedgehog Signaling to Promote Tumor Progression and Sorafenib Resistance in Hepatocellular Carcinoma

Contents：

Supplementary Figures: Pages 1-5

Supplementary Legends: Pages 6

Supplementary Figure: 1

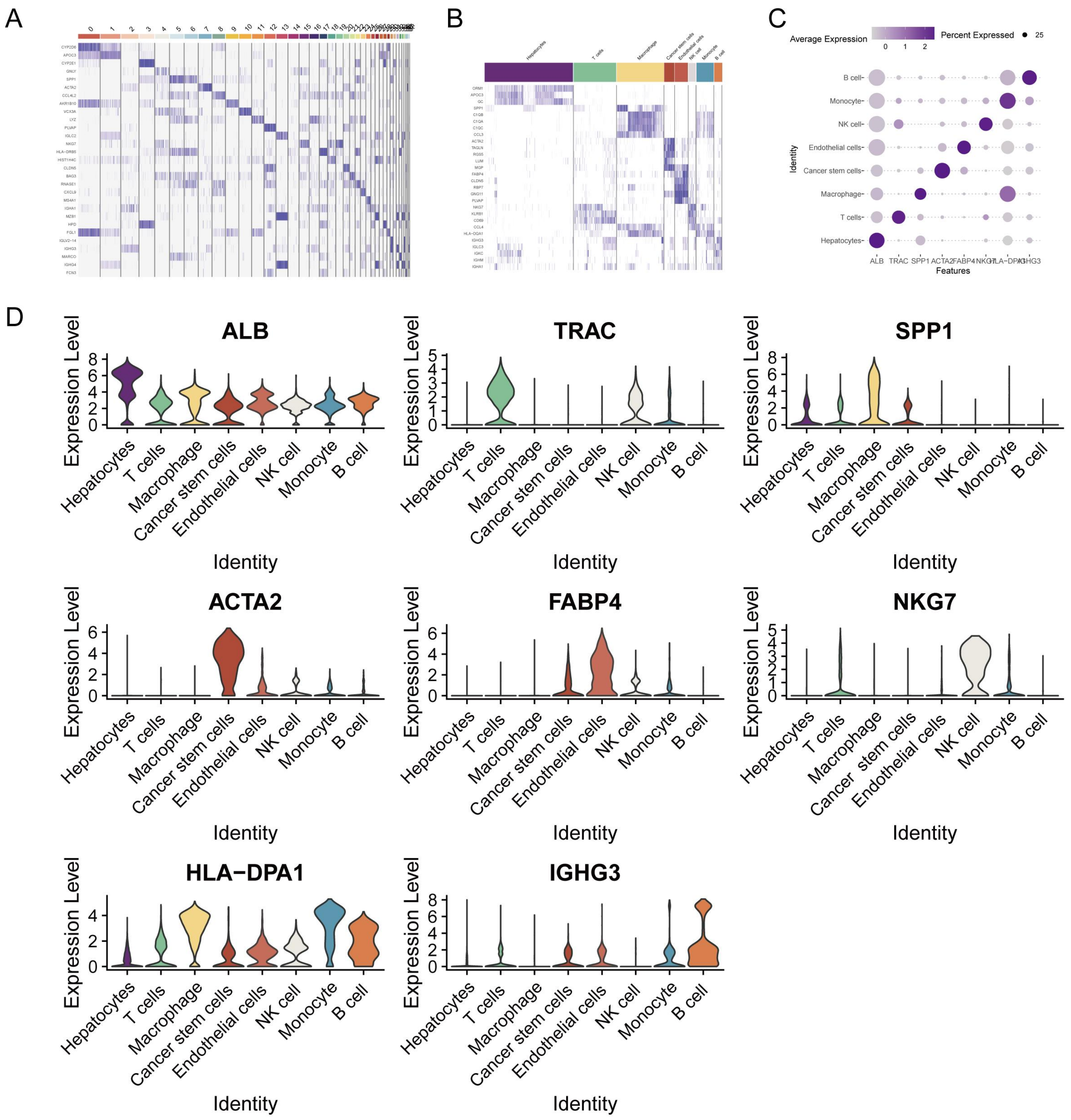

Supplementary Figure: 2

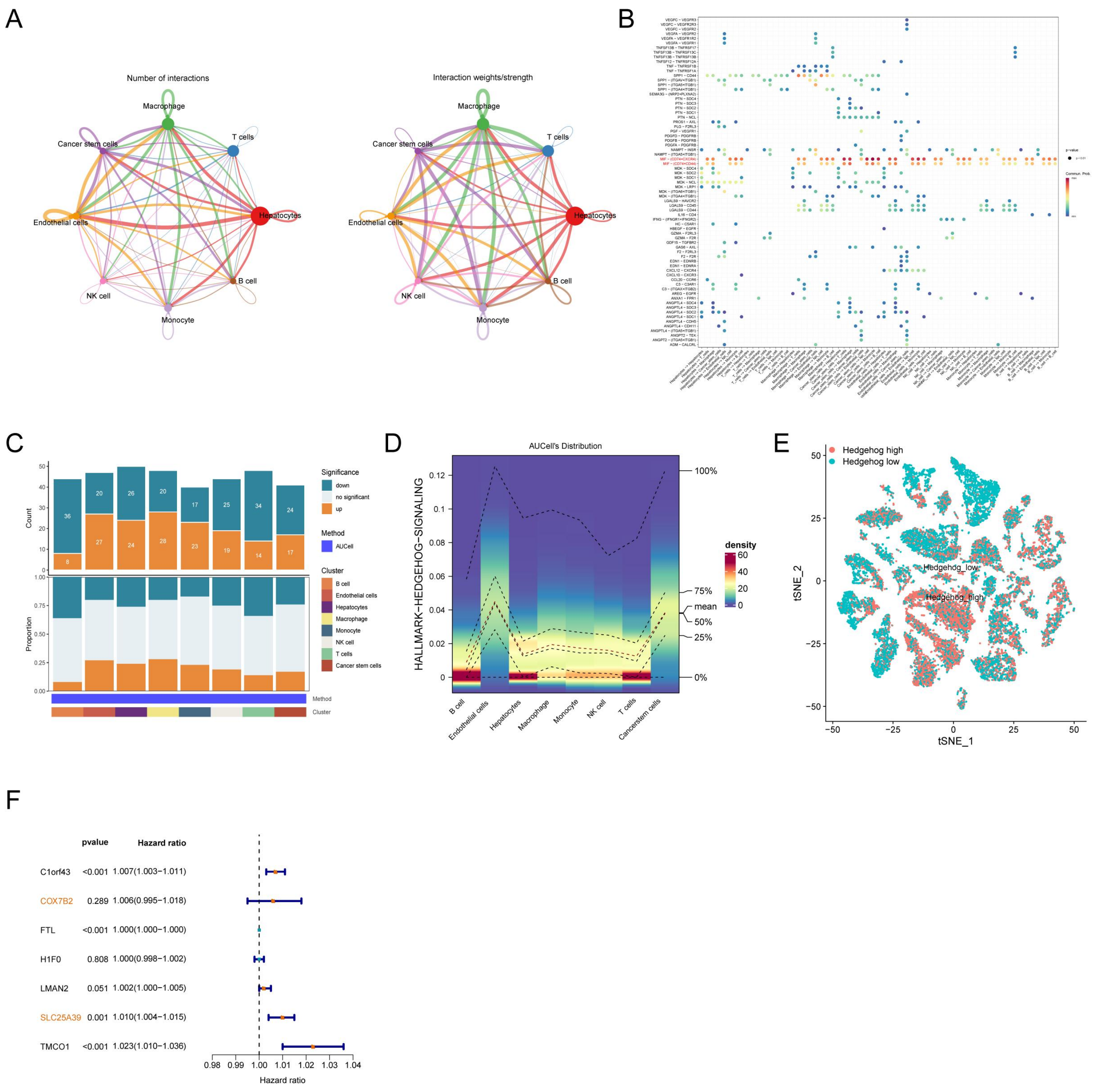

Supplementary Figure: 3

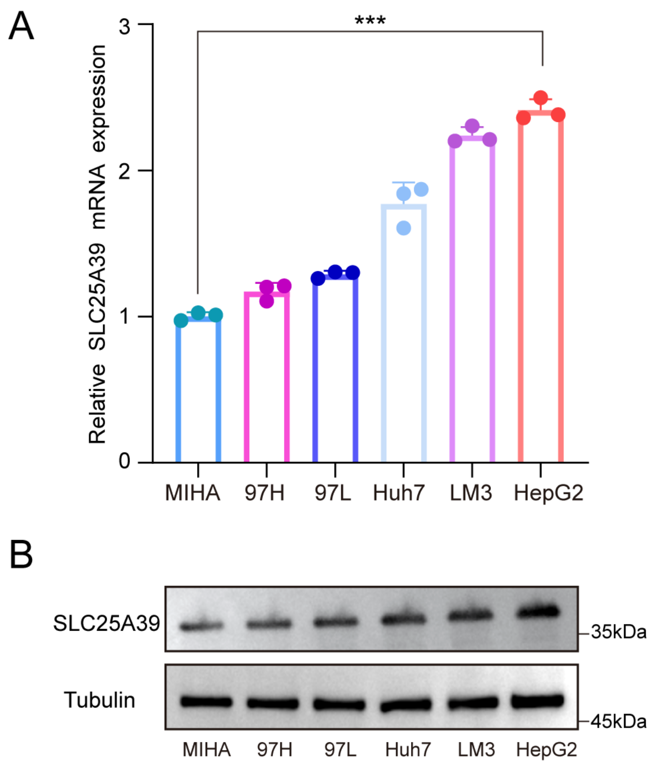

Supplementary Figure: 4

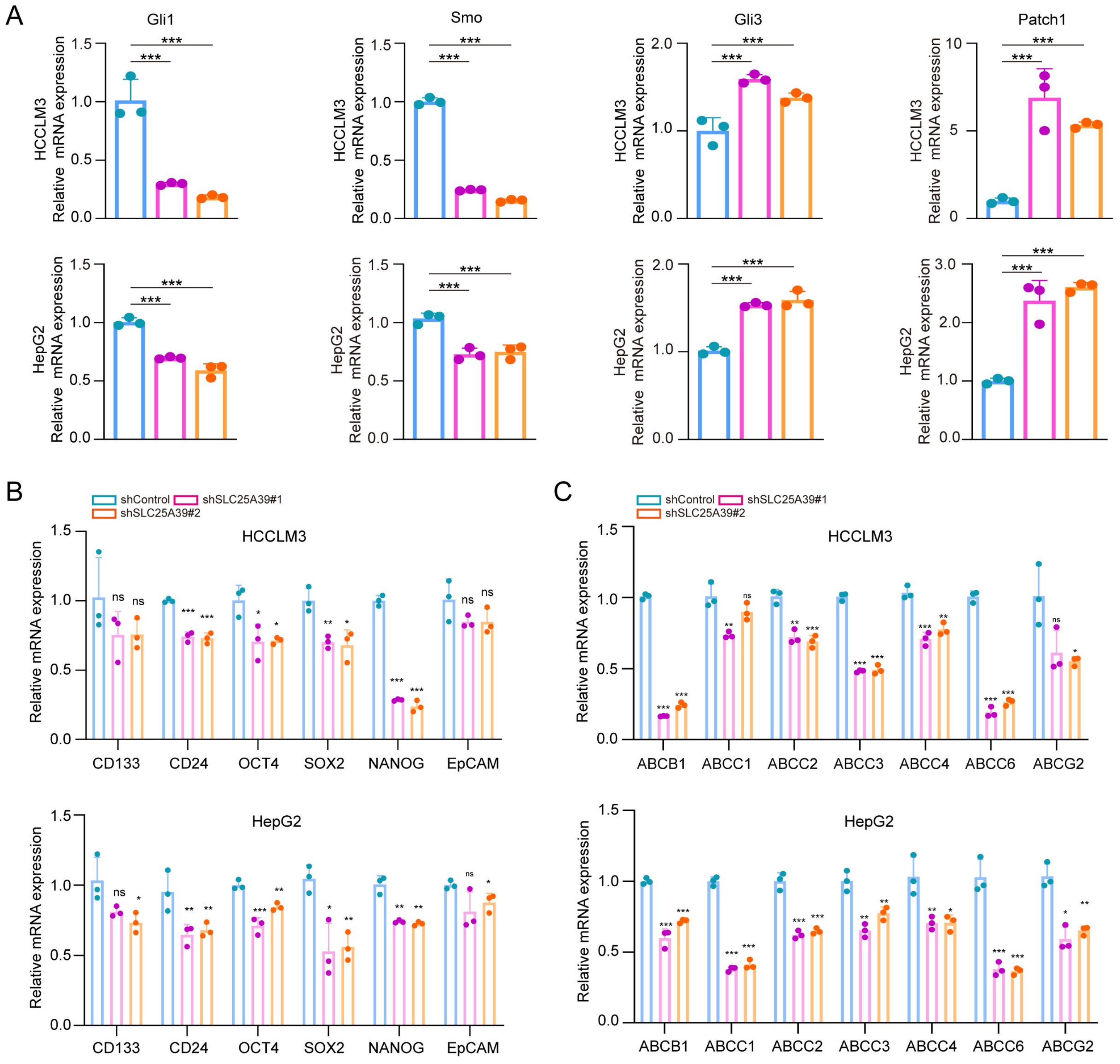

Supplementary Figure: 5

A

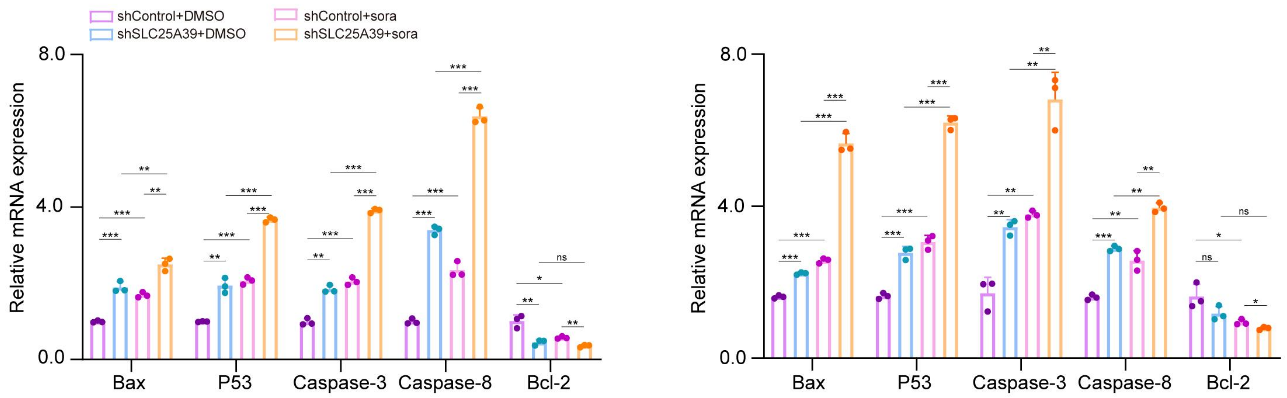

B

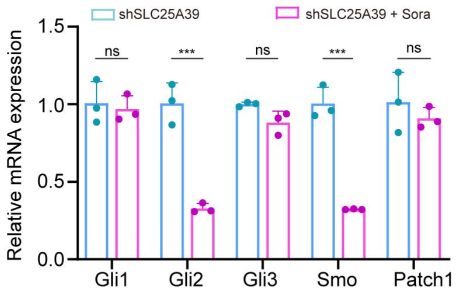

Supplementary Figure legends

Figure S1: (A) A heatmap displaying significantly differentially expressed genes across the clusters. (B) A heatmap illustrating significant differential gene expression among various cell types. (C) Bubble plots depict the expression levels of marker genes within each cell type. (D) Violin plots depict the expression levels of specific marker genes for each cell type.

Figure S2: (A) Count and statistics of enriched pathways for different cell types. (B) Scoring quantification of the Hedgehog pathway within cells using the AUCell function. (C) Hedgehog pathway scores for all cells categorized into high and low expression groups. (D) Visualization of the communication frequency and strength between the different cell types. (E) A bubble plot displaying the receptor-ligand molecules involved in cell communication. (F) A univariate Cox regression analysis was performed on the seven H-DEGs using data from the TCGA database.

Figure S3: (A) The expression levels of SLC25A39 mRNA in each cell line were detected by qRT-PCR. (B) The expression of SLC25A39 protein in each cell line was analyzed by Western Blot. Mean ± SEM. * $p < 0.05$, ** $p < 0.01$, *** $p < 0.001$.

Figure S4: (A) The mRNA expression changes of Hedgehog pathway-related genes after SLC25A39 knockdown were evaluated by qRT-PCR. (B) The mRNA expression levels of cancer stem cells (CSCs) marker genes after SLC25A39 knockdown were detected by qRT-PCR. (C) The mRNA expression levels of drug resistance genes after SLC25A39 knockdown were detected by qRT-PCR. Mean ± SEM. * $p < 0.05$, ** $p < 0.01$, *** $p < 0.001$.

Figure S5: (A) The mRNA expression of apoptosis-related genes after SLC25A39 knockdown and/or sorafenib treatment was examined by qRT-PCR. (B) The mRNA expression changes of Hedgehog pathway-related genes following the synergy between shSLC25A39 and sorafenib were evaluated by qRT-PCR. Mean ± SEM. * $p < 0.05$, ** $p < 0.01$, *** $p < 0.001$.
